# New wine in an old bottle? A facet-level perspective on the added value of Grit over BFI–2 Conscientiousness

**DOI:** 10.1371/journal.pone.0228969

**Published:** 2020-02-13

**Authors:** Fabian T. C. Schmidt, Clemens M. Lechner, Daniel Danner

**Affiliations:** 1 Leibniz Institute for Science and Mathematics Education, Kiel, Germany; 2 University of Hamburg, Hamburg, Germany; 3 GESIS Leibniz Institute for the Social Sciences, Mannheim, Germany; 4 University of Applied Labour Studies, Mannheim, Germany; Aalborg University, DENMARK

## Abstract

There is emerging consensus that Grit’s two facets—perseverance of effort and consistency of interest—are best understood as facets of the Big Five dimension of Conscientiousness. However, an in-depth investigation on whether Grit’s facet offer any added value over more established facets of Conscientiousness is absent from the literature. In the present study, we investigated whether Grit’s facets are empirically distinguishable from three facets of Conscientiousness as conceived in the well-validated Big-Five Inventory 2 (BFI–2), namely, Organization, Responsibility, Productiveness. Moreover, we investigated whether Grit’s facets show different (and possibly stronger) associations than the facets of Conscientiousness with a broad set of external criteria (age, educational attainment, monthly income, life satisfaction, mental and physical health, fluid and crystallized intelligence); as well as whether the criterion correlations of Grit’s facets are *incremental* over Conscientiousness. Findings from two latent-variable models in a large and diverse sample (*N* = 1,244) indicated that the facets of Grit showed moderate to strong relationships related to each other and to the three Conscientiousness facets of the BFI–2 (.41 ≤ *r* ≤ .94). Grit–Perseverance was almost indistinguishable from the Productiveness facet of Conscientiousness, whereas Grit–Consistency appeared to capture something unique beyond the Conscientiousness facets. The relationships with external criteria of Grit’s facets were similar in direction and size to those of the Conscientiousness facets. The results give further purchase to the view that Grit’s facets can be subsumed under the Conscientiousness domain.

Recently, Grit—defined as perseverance of effort and consistency of interest—has attracted considerable attention from both academic and lay audiences for its purported relevance for success in education and beyond. To be gritty means to stick to one’s long-term goals over months or even years, even when the going gets tough. Grit has been acclaimed as a key predictor of success, especially in academic settings (e.g., [1–4]). At the same time, critics have questioned the conceptual distinctness of Grit from the Big Five domain of Conscientiousness (i.e., the disposition to be organized, productive, and responsible), a construct well known for its utility to predict academic achievement as well [5]; and cast doubt on the alleged predictive power of Grit in relation to performance criteria [6, 7]. In addition, the close relationship between Grit and various measures of Conscientiousness has been discussed conceptually as well as empirically (e.g., [2, 7–10]).

Two main camps have emerged in the debate surrounding the added value of the Grit construct. The first of these camps views Grit as an incremental facet of Conscientiousness that is closely related to, but nevertheless conceptually and empirically distinct from, other facets [2, 4, 11]. As such, Grit might provide added value over established Conscientiousness facets in the prediction of important outcomes, such as GPA. The other camp has taken a more critical stance toward Grit, criticizing Grit as being merely a new label for (i.e., being indistinguishable from) Conscientiousness that fails to provide any added value [6, 7, 12, 13]—or in short, “old wine in a new bottle”. Only Grit’s perseverance of effort facet (henceforth Grit–Perseverance) but not the consistency of interest facet (henceforth Grit–Consistency) has been credited with bringing some added value to the field by this camp [7].

To contribute to this ongoing debate on the added value of Grit over Conscientiousness, we argue that a more nuanced facet-level perspective on both Grit and Conscientiousness is required. Accordingly, in the present study we investigated whether (1) the two facets of Grit, Grit–Perseverance and Grit–Consistency, are empirically distinct from other established facets of Conscientiousness; and (2) how the associations of Grit’s facets with a broad range of external criteria, including relevant success measures, compare to those of Conscientiousness facets with the same criteria. For this purpose, we selected two well-validated instruments to assess the two facets of Grit [1; German version, [14]; and the three facets of Conscientiousness distinguished by the Big Five Inventory–2 (BFI-2; [15]; German version, [16]). Paying heed to the faceted nature of both Grit and Conscientiousness helped us to overcome the shortcomings of previous studies that mostly used compound measures of Grit and Conscientiousness only; and to provide a more nuanced perspective on the relations of these constructs.

## Conceptual and empirical distinctions between Grit and Conscientiousness

In their initial publication introducing Grit, Duckworth et al. [1] described Grit as an achievement aspect of Conscientiousness. Still, they asserted the distinctness of Grit from Conscientiousness by stressing Grit’s focus on *long-term* stamina in the pursuit of personal goals, even in the face of obstacles––as opposed to more short-term aspects of self-regulation (e.g., temptation resistance) and the short-term intensity that dominate the most common conceptions of Conscientiousness [17]. Notwithstanding these authors’ assertion that Grit is distinct from Conscientiousness, a broad body of evidence attests to substantial associations between measures of the two constructs.

The most comprehensive study in this regard is the meta-analysis by Credé et al. [7], who found overall strong relationships (ρ = .84) between a variety of Conscientiousness measures and Grit. Based on their findings, these authors largely dismissed Grit, arguing that Grit was little more than a repackaging of Conscientiousness (see also [6] for a discussion). Moreover, they questioned the value of the two facets of Grit, arguing that the primary utility in the Grit concept lies in Grit–Perseverance because it is a stronger predictor of achievement than the Grit–Consistency facet or overall Grit. Abuhassàn and Bates [18] drew similar conclusions in their study on the relationships between the facets of Grit and a compound measure of Conscientiousness. They found Grit–Perseverance and Grit–Consistency to be closely, but differentially related, to Conscientiousness and other external criteria.

This empirical overlap is perhaps not surprising, as the conceptual similarities between Conscientiousness and Grit are substantial. This is especially true when taking a facet-level perspective. Grit shows particularly close definitional overlap with the Industriousness (or Productiveness) facet of Conscientiousness, as in-depth studies on the composition of Conscientiousness have shown [19, 20]. The same is true for some of the facets of commonly used comprehensive measures to assess Conscientiousness, such as the Achievement-striving and Self-discipline facets of Conscientiousness in the Revised NEO Personality Inventory (NEO-PI-R [21]; for a discussion see [8]), as well as the Productiveness and Responsibility facets of the Big Five Inventory–2 (BFI-2 [15]), which we use in the present investigation. Given the close relationships between Grit and particularly those facets of Conscientiousness that focus on proactivity and persistence, a view that has been espoused by several authors is that Grit should best be conceived of as facet of Conscientiousness, rather than as a measure for the broader Conscientiousness domain (e.g., [22]).

Even though the view that Grit represents a construct within—or a facet of—the Conscientiousness domain is an emerging consensus to which we subscribe in the present investigation, few studies have actually investigated the relationships between facets of Grit and facets of Conscientiousness (as opposed to domain-level measures of Conscientiousness) empirically. Apart from the study by Abuhassàn and Bates [18] named earlier, a recent study by Schmidt et al. [8] used a more comprehensive and broader measure with a focus on the industriousness aspects of Conscientiousness, the NEO-PI-R [21]. The study showed that Grit shared nearly 90% of its variance with Conscientiousness on the latent-variable level. However, on the facet level, the magnitude of associations varied considerably. While Grit–Perseverance shared most of its variance with the superordinate Industriousness and Conscientiousness factors (95%), Grit–Consistency showed overall weaker relationships with the superordinate factors (53%) but instead a significant relationship with the lower-level facet Self-discipline. This suggests that Grit–Perseverance and Grit–Consistency are differentially related to the Conscientiousness facets of the NEO-PI-R.

All in all, previous findings suggest that there are close conceptual and empirical relationships between Grit and Conscientiousness. Moreover, research suggests that the facets of Grit might best be viewed as facets of the Conscientiousness domain. However, whether Grit’s facet provide added value over the already established facets of Conscientiousness––especially over those facets such as Productiveness (or Industriousness) to which Grit is conceptually most closely related––remains a largely open question. In particular, previous studies have left largely unanswered the question as to whether Grit, Conscientiousness, and their respective facets have similar or different associations with external criteria.

In order to move the debate surrounding the added value of Grit over Conscientiousness forward, our present investigation approaches the question as to whether Grit provides added value over some aspects of Conscientiousness from a facet-level perspective on both constructs. This facet-level perspective has several advantages. In particular, it allows (1) to assess whether the two facets of Grit are distinct from established facets of Conscientiousness; and (2) to investigate whether Grit’s facets show similar or different associations with external criteria than the facets of Conscientiousness; as well as whether the associations of Grit with these criteria are incremental over those of Conscientiousness (i.e., persist after controlling for Conscientiousness). A recently developed and extensively (including cross-culturally) validated measure that allows to address these research questions and which we used in the present investigation is the BFI-2 [15], which we will introduce in the following.

## Locating Grit in the nomological network of BFI-2 Conscientiousness

As described above, the measures with which Conscientiousness can be assessed vary substantially and, thus, developing a clear rationale before selecting a measure is essential [20]. We applied the widely used Conscientiousness facets of the Big Five Inventory–2 (BFI-2 [15]; German version [16]). The facet structure of the BFI-2 can be seen as a synthesis of alternative Big Five facet structures (e.g., [23–25]) that covers a broad bandwidth of the Big Five domains in a parsimonious fashion. The BFI-2 assesses Conscientiousness with three facets, namely, Organization, Productiveness, and Responsibility.

Organization is defined as a preference for order and structure. It represents a largely inhibitory facet of Conscientiousness [26]. Organization and its conceptual equivalents from other faceted instruments, such as Order, Orderliness, or Tidiness, are commonly seen as the most central aspect of Conscientiousness [27], including by the authors of the BFI-2 [15]. It has been shown to represent a higher-order factor on the lowest level in the hierarchical Conscientiousness structure introduced by Roberts et al. [20] that emphasizes the ability to plan and to organize tasks and activities.

Productiveness, defined as a strong work ethic and persistence in pursuing goals, taps the more proactive aspect of Conscientiousness. As such, it is the conceptual equivalent to the Industriousness facet described in several in-depth studies of the structure of Conscientiousness [19, 20] and the Achievement-striving facet in the NEO-PI-R [21]. As mentioned above, NEO-PI-R Industriousness is closely related to Grit and shares nearly all of its variance with Grit–Perseverance [8].

Finally, Responsibility represents a person’s reliability and his or her commitment to fulfill duties and obligations [15, 20]. In other words, Responsibility captures the degree to which a person can be depended on. In its conception, Responsibility is likely to be most closely related to Grit–Consistency, as both facets represent a staying-on-course attitude towards goals. Grit–Consistency theoretically thus stands in closer relation to Responsibility than to the other facets of Conscientiousness that are assessed with the BFI-2.

In summary, the BFI-2 Conscientiousness measure with its three facets enabled us to investigate the extent to which Grit’s two facets are related to, or distinct from similar facets of Conscientiousness. If the view is correct that Grit can be conceived of as a facet of Conscientiousness––one that is distinct from other facets in that it puts particular emphasis on *long-term* persistence in goal pursuit [2]––then Grit–Perseverance and Grit–Consistency should be distinguishable from the three Conscientiousness facets of the BFI-2 (divergent validity). Contrariwise, if the view is correct that Grit is just “old wine in new bottles” [6, 8, 12], Grit’s two facets should be nearly indistinguishable from the three facets of Conscientiousness. Given their definitional overlap and the similarity of the items used to measure them (see S1 Table), we expected Grit–Perseverance to be most closely related to the Productiveness facet of BFI-2 Conscientiousness; and Grit–Consistency to be most closely related to the Responsibility facets of Conscientiousness. Just how close these relationships are was the first research question that we sought to answer in this study.

## The (incremental) criterion validity of Grit’s facets over Conscientiousness

In addition to establishing their divergent validity vis-à-vis other facets of Conscientiousness, an at least equally important way to judge the added value of Grit’s facets over those of Conscientiousness is whether Grit’s facets have incremental associations with important external criterion variables such as income, educational attainment, cognitive ability, or mental and physical health. If Grit’s facets show incremental associations with relevant criteria over the facets of Conscientiousness, this would bolster the view that Grit offers added value for the study of trait–outcome relations. Moreover, to the extent that Grit–Perseverance and Grit–Consistency show *differential* associations with at least some criteria, this would buttress the value of distinguishing these facets.

Both the incremental criterion validity of Grit over Conscientiousness and the value of distinguishing between Grit–Perseverance and Grit–Consistency have received at best mixed support by previous research. On the one hand, numerous studies have supported the predictive validity of Grit, particularly in relation to academic outcomes [1, 2, 11, 28–31]. In addition, some studies have suggested Grit’s incremental predictive validity over Conscientiousness for other domains such as career success, career engagement, and exercise behavior [10, 32–35]. On the other hand, a substantial number of studies found that Grit did not have any incremental predictive validity over Conscientiousness for a number of achievement outcomes such as GPA [7, 12, 13, 36–39]. In their recent meta-analysis, Credé et al. [7] conclude that either overall Grit nor Grit–Consistency added to the understanding or prediction of academic performance. These findings suggest that Grit does not add to the canon of constructs in psychology.

How can these diverging findings concerning the incremental criterion validity of Grit over Conscientiousness be explained? Most of the studies investigating the relative contribution of Grit towards the relationships with real-life outcomes have used rather short compound measures to assess Conscientiousness. One of the drawbacks of this approach is that short compound measures do not differentiate on the facet level and, thus, do not allow separating domain-general from facet-specific variance; such differentiation is a prerequisite for a clear interpretation of results when investigating the relationships of Grit and Conscientiousness with external criteria.

Furthermore, the definitions and conceptualizations of Conscientiousness varied widely across previous studies. This can lead to varying results depending on the measures used [19]. Previous studies such as the meta-analysis by Credé and colleagues [7] did not control for these variations in the measurement of Conscientiousness. In addition to variations in measurement, only few studies so far have investigated the predictive power of Grit on the facet level. Even in their initial study introducing the construct, Duckworth et al. [1] mostly used composite Grit scores without further differentiating its two facets. These studies commonly found Grit–Perseverance to have greater criterion validity than the Grit–Consistency facet [11, 18, 38, 40]. These findings again emphasize the importance of investigating the relationships between Grit and Conscientiousness on the facet level.

## The present study

In the present research, we aimed to shed further light on the question whether Grit provides any added value over Conscientiousness by taking a facet-level perspective. More specifically, we investigated (1) the associations between the two facets of Grit (Grit–Perseverance and Grit–Consistency) and the three facets of Conscientiousness in the BFI–2 (Organization, Productiveness, and Reliability) to test whether Grit’s facets are empirically distinguishable from those of Conscientiousness. Moreover, we (2) compared the strengths of the associations of the facets of Grit and Conscientiousness with a diverse set of external criteria: age, educational attainment, monthly income, life satisfaction, mental and physical health, and fluid (Gf) and crystallized (Gc) intelligence. We also tested whether Grit’s facets shows associations with these criteria over and above Conscientiousness. For this purpose, we modelled the Grit facets and the Conscientiousness facets as latent variables that are adjusted for measurement error.

We included a broad range of external correlates that were either used in previous studies on Grit and/or in the recent BFI-2 validation study [15]. The main purpose of including these correlates was to establish whether the facets of Grit and Conscientiousness would show differential relationships with these correlates. For this purpose, the direction of causal influence between Grit and the correlates was of minor import, and we were unable to establish causal effects with the cross-sectional design–a limitation our study shares with the bulk of previous research on Grit. That said, it seems very plausible that some of these correlates are more likely to reflect a causal influence of Grit than others, whereas other are more likely to reflect reciprocal influences, and yet others are mere correlates.

Income and crystalized intelligence (i.e., acquired knowledge) are likely to be the result of continued investment of time and effort. It is this type of success measure that most research on Grit has focused on (e.g., [1, 10, 32]). Whereas the positive relationships of Conscientiousness with income are well established [41–43], only few studies have investigated relationship between Grit and income, but the few studies that did generally found positive associations. For example Lechner et al. [10] found that grit was positively related to income even after controlling for Conscientiousness. Similarly, Danner et al. [44] found that Grit was linked to a higher income in most (but not all) of the 19 countries they studied. Hence, there is reason to believe that Grit is one of the prerequisites for earning a higher income. To the best of our knowledge, there is only one prior study testing the relationship between Grit and crystallized intelligence showing weak relationships [10] and the findings on the relationship between facets of Conscientiousness and crystallized intelligence vary substantially [45, 46]. To address this lacuna, we included a measure to assess crystallized intelligence in our study and investigated the relationships on the facet level for the first time. One could expect Grit could promote the acquisition of knowledge and skills (i.e., crystallized intelligence) by fostering sustained engagement and learning.

Life satisfaction, health, and educational attainment are likely to reflect reciprocal influences. For example, grit may foster educational attainment [2, 8], but educational institutions—especially those of higher education—are also likely to demand and foster Grit [47]. Research shows positive relationships between life satisfaction and Grit [48, 49] as well as between life satisfaction and Conscientiousness [43]. There is a broad body of research on the connection between Conscientiousness and health behavior or physical health (e.g., [50–52]. With regard to Grit, research is scarcer, yet some studies found positive relationships between Grit and physical health as well as fitness outcomes [53, 54].

Finally, for age and fluid intelligence, we assumed no causal influences of Grit or Conscientiousness, and previous research points to small and varied associations. Research has found that age is positively related to Grit (e.g. [2]) and Conscientiousness (for an overview, see [55]), although more recent findings suggest that the association between age and grit is an inverted u-shaped one [10].

Regarding fluid intelligence, the majority of previous studies found Grit to be largely independent of fluid intelligence and other measures of basic cognitive abilities [1, 12, 28, 32], a finding that has been meta-analytically confirmed [7]. Based on such findings, proponents of grit have claimed that Grit is a resource that could be fostered independently of cognitive ability—and that could be an equally—or even more—potent predictor of life success than cognitive ability [56]. However, it should be noted that few of these studies used pure measures of fluid intelligence, and those that did found small positive associations (e.g., [12]). Conscientiousness, on the other hand has mostly been shown to stand in a negative relationship with fluid intelligence [57, 58], except for its Responsibility facet [46]. To address this lacuna, we included a measure to assess fluid intelligence in our study and investigated the relationships on the facet level for the first time.

As stated above, we expected to find positive correlations between Grit and all of the external criteria––with the exception of fluid intelligence, from which Grit has repeatedly been found to be independent (e.g., [1, 7]). In light of the ongoing discussion on the relevance of Grit–Consistency and the empirical findings so far [7, 12], we expected Grit–Perseverance to show stronger relationships with the external criteria than Grit–Consistency. However, given the close definitional and item overlap between Grit–Perseverance and Productiveness as well as between Grit–Consistency and Responsibility, the crucial question was whether Grit’s facets hold any value when compared to those of Conscientiousness. This question of whether the relationships that Grit–Perseverance and Grit–Consistency have to external criteria were different from, or even stronger than, those that the three Conscientiousness facets have to these criteria was the second research question that we sought to answer in this study.

## Method

A list of all measures used, the data and the data analysis scripts needed to reproduce all of our reported results, and the results for Model B with Productiveness and Reliability as reference domains are open and available to download [59].

### Sample

We drew on a large and heterogeneous sample of German adults, recruited via an Online Access Panel in December 2016. This sample was quoted by age, gender, and education, in accordance with the German Census from 2011. Respondents with response times averaging below three seconds per item were excluded from further analyses (*n*_*excluded*_ = 124). Subjects have been properly instructed and have indicated that they consent to participate by agreeing to an appropriate informed digital consent form. The collection of data followed the technical and ethical standards of the Programme for the international Assessment of Adult Competencies. Strict anonymity was ensured, and participants cannot be uniquely identified. No specific ethical approval was necessary for this study because it did not involve potentially harmful stimuli. The final sample comprised *N* = 1,224 adults (*M*_*age*_ = 42.78; *SD*_*age*_ = 13.95; 50.4% female). The size of the sample was sufficiently large (i.e., *N* > 400) to reliably estimate models with modest factor loadings and relatively small effect sizes [60]. The items under study here were administered online as part of a larger study.

### Measures

#### Grit

We used six items of the German 9-Item Grit Scale [16], which is based on the work by Duckworth et al. [1] as well as that by Tangney, Baumeister, and Boone [61]. The reliabilities for all facets are reported in Table 1. These six items measured the Grit facets Grit–Perseverance (e.g., “I am a hard worker”) and Grit–Consistency (e.g., “New projects sometimes distract me from previous ones”) with three items each. Respondents answered these items on a five-point Likert-type scale ranging from 1 (*not at all*) to 5 (*to a very high extent*). S1 and S2 Tables (see Supplemental online material) provide the wording of all items in English and German, respectively.

**Table 1:** Latent correlations and reliabilities (ω) for the facets of Grit and Conscientiousness in the correlated first-order factors model (Model A).

	Correlations (Pearson’s *r*)				ω
	Consistency	Productiveness	Responsibility	Organization	
*Grit*					
Perseverance	.80	.94	.80	.52	.57
Consistency		.73	.81	.41	.65
*Conscientiousness*					
Productiveness			.91	.71	.73
Responsibility				.71	.60
Organization					.83

All correlations are statistically significant at *p* < .001.

#### Conscientiousness

We assessed Conscientiousness with the BFI-2 [15]; German version: [16]). The BFI-2 facets were constructed to strike a balance between bandwidth and fidelity. The aim was to represent the empirically most prominent, and clearly distinguishable, facets of each domain in a parsimonious fashion (for details on the construction rationale, see [15], p. 121). The authors proceeded by first selecting one “factor-pure” facet per domain that previous research identified as central to its own domain and as independent from the other four Big-Five domains; these factor-pure facets were used to construct the Big Five trait space. Two additional complementary facets were then added to each domain, which were identified from the personality literature and inventories. For Conscientiousness, a comprehensive literature review and existing facet models pointed to three key facets: Organization (or Orderliness), defined as preference for order and structure (e.g., “I am someone who is systematic, likes to keep things in order”); Productiveness (or Industriousness), defined as work ethic and diligence while pursuing goals (e.g., “I am someone who is efficient, gets things done”); and Responsibility, which stands for commitment to fulfilling duties and obligations (e.g., “I am someone who is reliable, can always be counted on”). Among these facets, Organization is the most factor-pure facet (it will also serve as the reference domain in our models; see Statistical Procedures). Each of these three Conscientiousness facets was measured by four items. The same five-point Likert-type scale as for Grit was used as the response format (1 = *I do not agree at all* to 5 = *I fully agree*).

#### External criteria

We selected a broad variety of external criteria in order to gauge the power of Grit over Conscientiousness. Fluid intelligence (Gf) was assessed using the short form of the Hagener Matrices Test (HMT-S; [62]). The test comprises six items measuring fluid reasoning according to the Cattell-Horn-Carroll model of intelligence [63]. Respondents had two minutes to complete each item. To assess crystallized intelligence (Gc), we used a short form of the crystallized intelligence subscale of the Berlin test for assessing crystallized and fluid intelligence (Berliner Test zur Erfassung Fluider und Kristalliner Intelligenz, BEFKI GC; [64]). The scale comprises 12 multiple-choice questions assessing declarative knowledge from areas such as science and the social sciences. Respondents had five minutes to complete the questionnaire.

Respondents indicated their educational attainment on a scale ranging from 1 = *no degree* to 6 = *university degree*. To measure monthly income, respondents indicated their gross monthly earnings on a 17-point ordinal scale (1 = *below €300* to 17 = €*10*,*000 and above*). Life satisfaction was assessed using one item: “All in all, how satisfied are you with your life at the moment?”. Respondents answered on a scale ranging from 1 = *entirely unsatisfied* to 11 = *entirely satisfied*. Finally, respondents’ current mental and physical health status was measured with one item: “*In general*, *how would you describe your (physical and mental) health*?” Responses were given on a five-point Likert-type scale (1 = *bad* to 5 = *very good*).

### Statistical procedures

We approached our research questions from the perspective of two structural equation models (SEM): a correlated first-order factor model (Model A) and a bifactor-(*S*–1) model (Model B; [65]). Each of these models provides unique, yet complementary, insights the relationships between the facets of Grit and those of Conscientiousness based on different assumptions. We will present these two approaches in more detail in the following.

#### Model A: Correlated first-order factors

**Fig 1:**
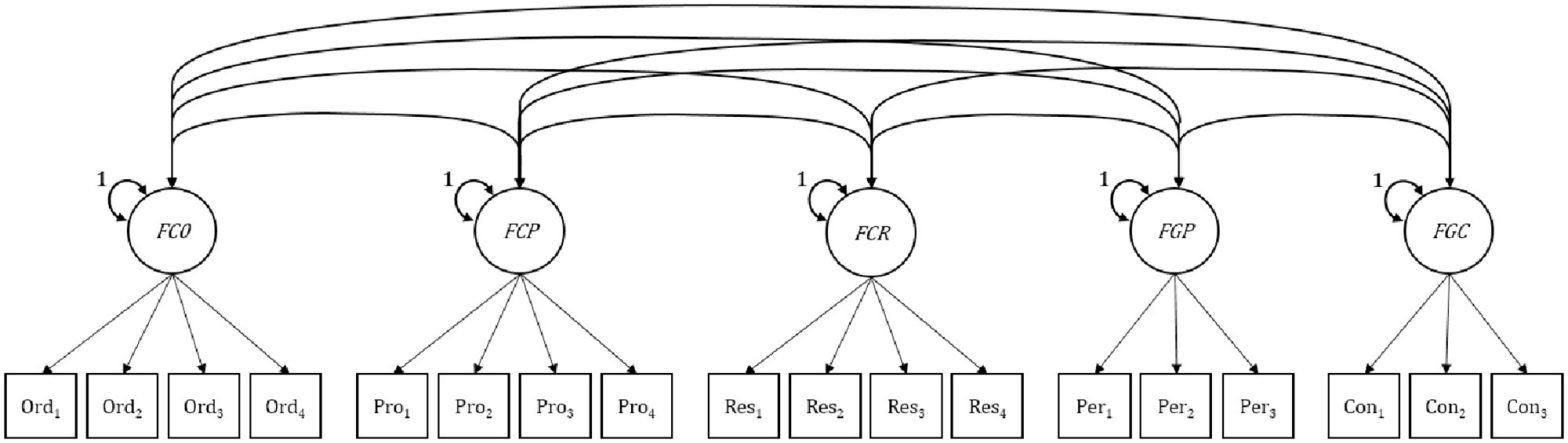
Correlated first-order factors model (Model A). Conscientiousness facets: FCO = Organization factor, FCP = Productiveness factor, FCR = Responsibility factor. Grit facets: FGP = Perseverance of effort factor, FGC = Consistency of interest factor. Latent acquiescence response style variable not depicted.

In Model A (see Fig 1), the two facets of Grit and the three facets of Conscientiousness were all modeled as correlated first-order factors without imposing any constraints (apart from the independent-cluster model assumption that there are no cross loadings). This model allowed us to compare the facets of Grit and Conscientiousness head to head by estimating the latent-variable correlations between all five facets, as well as their correlations with the external criteria.

In Model A, we judged the added value of Grit over Conscientiousness based on four criteria. We judged construct validity mainly in terms of divergent validity, testing (1) whether the model fit to the data without any cross-loadings or residual correlations; (2) whether the correlations among the Grit and the Conscientiousness facets, as well as between the two Grit facets, were low enough to suggest a minimum degree of empirical distinctness of the constructs, for which we set a benchmark value of < 90% shared variance, the equivalent of *r* < .95. Moreover, we judged the (incremental) criterion validity based on whether (3) Grit’s facets showed differential relationships to the external criteria, which would further buttress the distinction between Grit–Perseverance and Grit–Consistency; and (4) whether Grit’s facets show differential, or even stronger, relationships to the external criteria when compared to the three Conscientiousness facets.

#### Model B: Bifactor-(S–1) model

**Fig 2:**
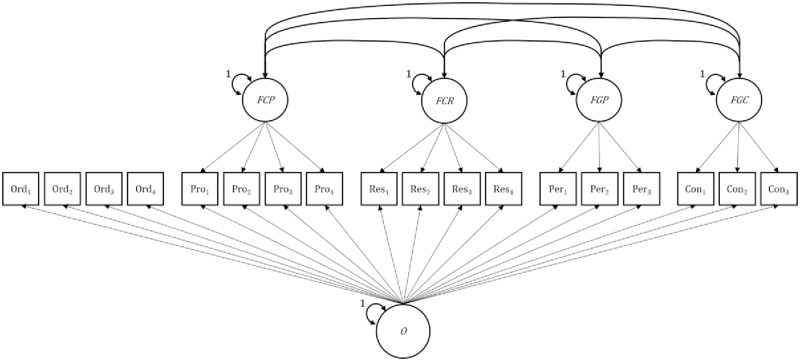
Bifactor-(*S*–1) model (Model B). O = Organization (Reference facet), FCP = specific Productiveness factor, FR = specific Responsibility factor, FGP = specific Perseverance factor, FGC = specific Consistency factor. Latent acquiescence response style variable not depicted.

Model B (see Fig 2) provides an alternative, and arguably more conservative, test of the added value of Grit over Conscientiousness. Instead of comparing all five facets head to head, it accounts for the fact that all facets of Grit and Conscientiousness share a substantial portion of their variance. The bifactor-(*S*–1) model achieves this by modelling a general (*g*) factor and a number of specific factors. In contrast to the traditional bifactor model, one specific factor is omitted, which changes the meaning of the *g* factor to a *reference* factor. Thus, Model B comprised one reference facet and four (i.e., *S–1*) specific facets.

The reference facet factor represents individual differences on the facet selected as the reference facet. The specific facets are modeled as residual factors (they are residualized with respect to the reference facet) and contain only the unique portion of variance of the facet that is not shared with the reference facet. Being residual factors, the specific facets are orthogonal to (i.e., uncorrelated with) the reference facets but can correlate with each other (in contrast to the traditional bifactor model, where they are uncorrelated). Residualizing them for the reference facet also changes the meaning of the correlations among the non-reference facets compared to Model A: The correlations between the specific facets in the bifactor(S–1) model and between these facets and exogenous criterion variables are part (semi-partial) and indicate whether the non-reference facets contain variance that is not shared with the reference facet, but that is shared with each other or with the criteria.

For this model, one facet of Conscientiousness needed to be chosen as the reference facet. Based on theoretical and pragmatic considerations, we chose Conscientiousness–Organization as the reference facet. Organization is commonly considered to be the most central aspect of Conscientiousness [15, 27] and constitutes the most “factor-pure facet of the domain” ([15], p. 121). In addition, choosing Organization as the reference facet allowed us to investigate the relationships between the theoretically most closely related facet pairs of Grit and Conscientiousness in order to examine the extent to which they are distinct: of Grit–Perseverance with Productiveness; and of Grit–Consistency with Responsibility. Likewise, our approach allowed us to compare the relationships of these closely related residual facet pairs with the external criteria.

The two non-reference facets of Conscientiousness (i.e., Productiveness and Responsibility) and the two Grit facets were modeled as specific (or “residual”) factors with means fixed to zero. These residual facets represent the incremental aspect of each facet that is not explained by the reference facet Organization. The correlations between the residual facets represent partial correlations corrected for the common influences of the Organization reference facet. All factors were identified by fixing the loading of the first item (λ = 1).

In Model B, we again judged the added value of Grit over Conscientiousness in terms of divergent validity based on whether (1) the residual Grit facets captured an incremental, and large-enough, portion of variance beyond the reference facet Organization. Apart from whether each residual facet possesses statistically significant variance (which is not always the case in applications), a key indicator of the relevance of a residual facet in the bifactor-(S–1) model is the specificity coefficient that Eid et al. [65] refer to as substantial when exceeding the threshold of *Spe*(τ_*ik*_) > .40. The specificity coefficient reflects the share of variance in the systematic variance of an item τ_*ik*_ that can be uniquely attributed to a residual factor ζ_*ik*_ (e.g., the residual Grit–Consistency factor) and is not shared with the reference factor (here, Organization). It is calculated as

$$Spe(\tau_{ik})=\frac{\lambda_{ik}^{2}Var(\zeta_{ik})}{Var(\tau_{ik})}$$

whereby λ_*ik*_ refer to the loadings on the residual facet factor and *Var*(ζ_*ik*_) refers to the variance of that factor. We also tested (2) whether the correlations among the non-reference (i.e., residual) Grit and Conscientiousness facets, as well as between the two Grit facets, were low enough to suggest empirical distinctness of the constructs, for which we again adopted a benchmark value of < 90% shared variance (i.e., *r* < .95). Moreover, we again judged criterion validity based on (3) whether Grit’s facets showed differential relationships to the external criteria; and (4) whether—even after accounting for their shared variance with the Organization reference facets—Grit’s facets showed differential, or even stronger, relationships to the external criteria compared to the Conscientiousness reference and residual facets.

#### Controlling for acquiescence

Acquiescent responding (“yeah saying”) is a major source of bias and model misfit in personality questionnaires [66]. In both Model A and B, we therefore controlled for acquiescent responding by modeling a latent acquiescence factor [67, 68]. This was possible because the scales comprised both positively and negatively keyed items. This latent acquiescence factor was specified to load positively on the positively keyed (all λ = 1) and negatively on the reverse-keyed (and prior to the analyses recoded) items (all λ = –1). This factor thus captures respondents’ tendency to agree with all items irrespective of content and keying and removes it from the substantive facet factors, with which it is uncorrelated.

#### Estimation

All models were estimated using M*plus*, Version 7.4 [69]. We used the means and variance adjusted weighted least squares estimation (WLSMV) estimator because our variables comprised almost exclusively categorical and only few continuous indicators. Several indices of fit have been suggested to evaluate the goodness of fit for SEM (e.g., [70, 71]). For the present analyses, we used the Comparative Fit Index (CFI), the Tucker-Lewis Index (TLI), the Root Mean Square Error of Approximation (RMSEA), and the Standardized Root Mean Square Residual (SRMR). CFI and TLI values greater than .90 are typically interpreted to reflect an acceptable or excellent fit to the data. RMSEA values lower than .08 and SRMR values lower than .08 are typically interpreted to reflect a close or a reasonable fit to the data [70, 71].

## Results

We proceeded in two steps. In the first step, we investigated the associations between the latent Grit and Conscientiousness facets from the perspectives of Model A and Model B, respectively. This allowed us to evaluate the construct validity of the Grit facets relative to the BFI-2 Conscientiousness facets. In the second step, we investigated the relationships of the facets as conceived in Models A and B with the external criteria, which allowed us to evaluate the incremental criterion validity of the Grit facets.

### Locating Grit in the Conscientiousness domain

#### Model A: Correlated first-order factor model

The correlated first-order factor model (Model A) showed good fit to the data, χ^2^(124) = 453.56, *p* < .001, CFI = .94, TLI = .93, RMSEA = .05 SRMR = .04. All factor loadings were substantial (.37 ≤ λ ≤ .80). The correlations between the first-order facet factors are reported in Table 1 along with their reliabilities. The means and standard deviations as well as all of the factor loadings are reported in Table 2. The factor loadings demonstrated that all item measure the constructs as intended (.37 ≤ *λ* ≤ .80).

**Table 2:** Descriptive statistics and standardized factor loadings for Model A and Model B (standard errors are given in parentheses).

				Model A	Model B	
Latent Variable	Item No.	*M*	*SD*	Loadings on first-order factors	Loadings on reference factor (Organization)	Loadings on the specific factors
*Grit*						
Perseverance	1	3.88	0.82	.73 (.02)	.37 (.03)	.64 (.03)
	2	3.19	0.91	.40 (.03)	.16 (.03)	.37 (.03)
	3	3.44	0.92	.37 (.03)	.16 (.03)	.34 (.03)
Consistency	4	3.50	0.91	.59 (.03)	.21 (.03)	.57 (.03)
	5	3.21	0.87	.51 (.03)	.29 (.03)	.41 (.03)
	6	3.51	1.06	.66 (.03)	.27 (.03)	.61 (.03)
*Conscientiousness*						
Productiveness	7	3.27	1.11	.62 (.03)	.53 (.02)	.32 (.03)
	8	3.20	1.07	.68 (.03)	.50 (.02)	.44 (.03)
	9	3.55	0.89	.61 (.03)	.46 (.03)	.40 (.03)
	10	3.95	0.81	.69 (.03)	.40 (.03)	.61 (.02)
Responsibility	11	3.62	0.80	.43 (.03)	.32 (.03)	.29 (.03)
	12	3.39	0.97	.67 (.03)	.50 (.02)	.43 (.03)
	13	4.36	0.72	.49 (.03)	.32 (.03)	.39 (.03)
	14	3.69	1.02	.50 (.03)	.33 (.03)	.41 (.03)
Organization	15	3.42	1.15	.80 (.02)	.80 (.01)	-
	16	3.52	1.02	.78 (.02)	.78 (.01)	-
	17	3.93	0.87	.68 (.02)	.67 (.02)	-
	18	3.83	1.11	.80 (.02)	.80 (.02)	-

As expected from the fact that all facets are part of a wider Conscientiousness domain, all correlations between the facets in Table 1 were positive, statistically significant, and large in size (.41 ≤ *r* ≤ .94). The two facets of Grit were closely related, yet the size of the correlation was still far from unity. Their correlation implied that the two Grit facets shared 64% (.80 × .80 = .64) of their variance, which indicates a sufficient degree of separation between the facets.

In terms of their correlations with the facets of Conscientiousness, the strongest correlation emerged between Grit–Perseverance and Productivity on the one hand and between Grit–Consistency and Responsibility on the other. These correlations implied that Grit–Perseverance and Productiveness shared 88% of their variance, whereas Grit–Consistency and Responsibility shared 66% of their variance. This suggests that Grit–Perseverance and Productiveness are closely related, almost to the point of being empirically indistinguishable, whereas Grit–Consistency is clearly distinct from Responsibility (as well as all other Conscientiousness facets). The correlations with the other facets of Conscientiousness were still substantial but considerably lower, with both Persistence and Grit–Consistency exhibiting their smallest relationship with Organization. This pattern of results is in line with our assumption that the facet pairs Grit–Perseverance and Productiveness, as well as Grit–Consistency and Responsibility, are theoretically most closely related. Overall, Grit–Consistency was more distinct from the three facets of Conscientiousness than Grit–Perseverance was.

#### Model B: Bifactor-(S–1) model

The bifactor-(*S*–1) model with Organization as the reference facet of Conscientiousness also showed a good fit to the data: χ^2^(114) = 441.18, *p* < .001, CFI = .96, TLI = .94, RMSEA = .05, SRMR = .03. The loadings of all items on the Organization reference factor (.16 ≤ λ ≤ .80) and of each facet’s items on its respective residual facet factor (.29 ≤ λ ≤ .64) can be found in Table 2.

The bifactor-(*S*–1) model made it possible to estimate the specificity coefficients, that is, the proportion of specific variance in a non-reference facet that is not shared with the reference facet [60]. The specificity coefficients for the four residual facets are reported in Table 2. The two residual Grit facets (Grit–Perseverance and Grit–Consistency) showed substantially higher specificities than the two residual Conscientiousness facets (Responsibility and Productiveness).

This means that the Grit facets shared less variance with the reference facet of Organization, which accounted for around 20% of the total variance in the Grit items compared to 54% for the Responsibility items and 60% of the Productiveness items. These results show that the residual Grit facets still capture a substantial amount of unique variance even after accounting for their overlap (i.e., shared variance) with the reference facet Organization.

The correlations among the specific (residual) facets can be found in Table 3 (recall Organization, the reference facet, is uncorrelated with all specific facets). The partial correlations between these residual facets were generally smaller than those of the first-order facets in Model A because all the common variance these facets shared with the Organization reference facet was already partialed out of the residual variables. However, even after adjusting for Organization, there was a high partial correlation of *r* = .95 between the residual facets of Productiveness and Grit–Perseverance, indicating that these residual facets shared 90% of their variance beyond the reference facet, to the point where they were empirically indistinguishable. Residual Grit–Consistency again had its closest relationship with residual facet Responsibility (*r* = .79), indicating that that these facets shared 62% of their incremental variance beyond the reference facet. Thus, as expected, and akin to Model A, the two residual facet pairs Productivity and Grit–Perseverance as well as Responsibility and Grit–Consistency showed the strongest relationships.

**Table 3:** Latent correlations and specificity coefficients (Spe) for the facets of Grit and Conscientiousness in the bifactor-(S–1) model with organization as the reference facet (Model B).

	Correlations w/				*Spe*
	Consistency (S)	Productiveness (S)	Responsibility (S)	Organization (R)	
*Grit*					
Perseverance (S)	.74	.95	.71	.00	.84
Consistency (S)		.69	.79	.00	.80
*Conscientiousness*					
Productiveness (S)			.79	.00	.40
Responsibility (S)				.00	.46

All freely estimated correlations are statistically significant at *p* < .001. Organization is modeled as the reference facet (R) and its correlations to the specific (or “residual”) facets (S) are fixed to zero. *Spe* denotes the specificity coefficient (i.e., the share of variance in the indicators explained by a residual facet

### The incremental criterion correlations of Grit’s facets beyond those of Conscientiousness

In this second part of the results section, we report the correlations with the external criteria: age, educational attainment, monthly income, life satisfaction, mental and physical health, and fluid and crystallized intelligence. The correlations estimated based on Model A revealed the extent to which the first-order Grit and Conscientiousness facets were associated with these external criteria in a head-to-head comparison. The correlations estimated based on Model B revealed the extent to which the residual facets were *incrementally* associated with the criteria beyond the reference facet Organization. The correlations for Model A and Model B are presented in Tables 4 and 5, respectively. The descriptive statistics and the intercorrelations of the external criteria are reported in S3 Table in the supplemental online material.

**Table 4:** Correlations of the facets of Grit and Conscientiousness with age, education, income, life satisfaction, health, and fluid and crystallized intelligence from Model A (correlated first-order facet factors).

	Age	Education	Income	Life satisfaction	Health	Fluid Intelligence	Crystallized intelligence	$\left|\bar{r}\right|$
*Grit*								
Perseverance	.12**	.12**	.22**	.30**	.29**	–.02	.12	.17
Consistency	.26**	.12**	.16**	.30**	.23**	.11	.30**	.21
*Conscientiousness*								
Productiveness	.22**	–.01	.17**	.25**	.21**	–.16*	–.08	.16
Responsibility	.32**	.05	.11**	.28**	.19**	–.06	.19*	.17
Organization	.20**	–.05	.10**	.15**	.09**	–.23**	–.14*	.14

*p < .05,

**p < .001.

Strongest correlation per outcome in bold face.

**Table 5:** Correlations with age, education, income, life satisfaction, health, and fluid and crystallized intelligence for Model B (organization as a reference facet).

	Age	Education	Income	Life satisfaction	Health	Fluid Intelligence	Crystallized intelligence	$\left|\bar{r}\right|$
*Grit*								
Perseverance (S)	.05	.19**	.23**	.31**	.34**	.15	.25*	.22
Consistency (S)	.18**	.16**	.12**	.26**	.20**	.22*	.37**	.22
*Conscientiousness*								
Productiveness (S)	.12**	.04	.14**	.21**	.21**	.07	.07	.12
Responsibility (S)	.28**	.13*	.05	.26**	.18**	.14	.44**	.21
Organization (R)	.19**	–.05	.10**	.14**	.09**	–.24**	–.15 *	.14

*p < .05,

**p < .001.

In Model B, Organization is modeled as the reference facet (R) and its correlations to the specific (or “residual”) facets (S) are fixed to zero. Strongest correlations per outcome in bold face.

#### Correlated first-order factor model

In Model A, the correlations with age were all small but statistically significant (.12 ≤ *r* ≤ .32), indicating that higher age was associated with slightly higher values on all facets of Grit and Conscientiousness (see Table 4). Grit–Perseverance had the smallest association with age, whereas Responsibility had the highest.

Educational attainment showed a more differentiated pattern of relationships with the five first-order facets (.05 ≤ *r* ≤ .12). Only the two the facets of Grit, but not those of Conscientiousness, showed statistically significant, positive correlations with educational attainment. The relationship did not differ between the two Grit facets. All correlations with monthly income were positive and statistically significant (.10 ≤ *r* ≤ .22). Grit–Perseverance and Productiveness exhibited the strongest associations with income, followed by Grit–Consistency. The correlational patterns for life satisfaction (.15 ≤ *r* ≤ .30) and health (.09 ≤ *r* ≤ .29) resembled one another. The two most closely related facet pairs (Grit–Perseverance and Productiveness; Grit–Consistency and Responsibility) showed the strongest correlations with life satisfaction and health. Fluid intelligence was the first criterion that was not correlated with both first-order Grit facets (–.23 ≤ *r* ≤ .11). Only the negative correlations with the first-order Organization and Productiveness facets were statistically significant. Finally, crystallized intelligence showed a broad range of relationships with the first-order Conscientiousness and Grit facets (–.14 ≤ *r* ≤ .30). Similarly to fluid intelligence, the first-order Organization facet was correlated negatively with crystallized intelligence. Interestingly, the two closely related facets, Responsibility and Grit–Consistency, were both correlated positively with crystallized intelligence, even though the relationship with Grit–Consistency was stronger. Again, Grit–Consistency and Grit–Perseverance showed a differential pattern of correlations.

Overall, the results from Model A suggest that the facets of Grit had mostly similar relationships to external criteria as the three facets of Conscientiousness, with the exception of a few more marked differences for single criteria. No facet among the five facets under study had consistently stronger associations with the criteria than the others. As the average absolute correlations ($\left|\bar{r}\right|$) per facet in the last column of Table 4 suggest, the two facets of Grit and the three facets of Conscientiousness did not differ markedly from each other.

#### Bifactor-(S–1) model

Now we turn to Model B to examine the extent to which the residual facets were incrementally associated with the external criteria above and beyond the reference facet Organization (see Table 5). Compared to Model A, associations were more varied. Age was positively related to all facets but Grit–Perseverance (.05 ≤ *r* ≤ .28), meaning that Grit–Perseverance showed no incremental association with age beyond that of Organization. For educational attainment (–.05 ≤ *r* ≤ .19), the pattern of relationships differed substantially. We did not find a statistically significant relationship between the reference facet and educational attainment. However, residual Grit–Perseverance, Grit–Consistency, and Responsibility showed incremental relationships over the reference facet. These results show that the residual Grit facets and Responsibility facet are incrementally associated with educational attainment over general Conscientiousness.

The correlations of monthly income (.05 ≤ *r* ≤ .23) with the reference and the specific facets were statistically significant. The residual Responsibility facet was the only residual facet that did not show an incremental relationship with monthly income. The most pronounced incremental relationship was found for residual Grit–Perseverance. The residual Grit–Consistency and Productiveness facets showed lower but similar incremental relationships with monthly income. All in all, the results show that both residual Grit facets and the residual Productiveness facet of Conscientiousness were related to monthly income over the reference facet.

Again, the correlations for life satisfaction (.14 ≤ *r* ≤ .31) and health (.09 ≤ *r* ≤ .34) resembled one another. For both external criteria, the reference facet Organization showed a positive, statistically significant relationship. In both cases, all residual facets showed incremental value over the reference facet. Residual Grit–Perseverance showed the strongest incremental relationship with life satisfaction and health.

Fluid intelligence was correlated negatively with the reference facet (–.24 ≤ *r* ≤ .22). Only residual Grit–Consistency showed a positive, incremental relationship with fluid intelligence. That pattern changed substantially for crystallized intelligence (–.15 ≤ *r* ≤ .44). Even though the reference facet Organization was negatively and statistically significantly correlated, again, not only residual Grit–Consistency but also the closely related residual Responsibility facet showed strong positive relationships with crystallized intelligence over the reference facet. In addition, residual Grit–Perseverance was related incrementally with crystallized intelligence as well, but to a lesser extent.

All in all, results from Model B demonstrate that the two residual Grit facets showed substantial relationships with the external criteria even beyond the reference facet Organization. These associations were mostly as large, or larger, than those we found in Model A, where these facets were modeled as first-order factors. It was only for the external criteria of age and fluid intelligence that the relationships with residual Grit–Perseverance did not reach statistical significance. Again, none of the facets emerged as clearly superior in terms of the strength of its associations across all criteria. However, as in Model A, Grit’s facets tended to be slightly more strongly related to those criteria indexing long-term life success (i.e., income, educational attainment, life satisfaction, health, and crystallized intelligence) than the facets of Conscientiousness. Akin to Model A, Grit–Consistency had equally strong associations with the external criteria as Grit–Perseverance on average across all external criteria. Moreover, Grit–Perseverance and Grit–Consistency both had mostly equally strong relationships to the external criteria as their most closely related counterparts among the Conscientiousness facets, Productiveness and Responsibility.

## Discussion

Is Grit a useful addition to existing measures of Conscientiousness that adds to our understanding of real-world success and performance; is it “new wine” wrongfully put “in an old bottle“; or is it just “old wine in a new bottle” that fails to provide any added value? In order to contribute to this debate revolving around the added value of the Grit construct, our present study took a facet-level perspective. Our point of departure was the view recently endorsed by several researchers that the facets of Grit should best be conceived as facets of the Big Five domain of Conscientiousness. To test whether this view stands a rigorous empirical test, we investigated (1) the relationships between the two facets of Grit (Grit–Perseverance and Grit–Consistency) and the three facets of Conscientiousness (Organization, Productiveness, and Responsibility) as conceived in the BFI-2. Moreover, we investigated (2) the criterion correlations of the Grit and Conscientiousness facets in relation to a broad range of criteria (age, educational attainment, monthly income, life satisfaction, health, as well as fluid and crystallized intelligence). By doing so, we addressed the two core questions raised in recent research on Grit, which are the touchstones for judging the utility and added value of the Grit construct: (How) can Grit and Conscientiousness be differentiated from other facets of Conscientiousness (if at all)? And to what extent does Grit have incremental value over Conscientiousness facets in regards to external criteria? We addressed these questions on the facet level and through two statistical models: a correlated first-order factors model (Model A) comparing the latent variables head-to-head; and a *bifactor-*(*S–1*) model (Model B) that enabled us to explicitly model the hierarchical structure of Conscientiousness, conceiving of Grit’s facets as specific (residual) facets of Organization, which is the most factor-pure facet of Conscientiousness ([15], p. 121) and served us as the reference facet. For our analyses, we used a large and heterogeneous sample of adults that was quoted in accordance with the German Census from 2011.

Three key findings emerged from both models. First, Grit’s two facets are related to all the three facets of Conscientiousness in the BFI-2, as one would expect if all five facets are conceived of as facets of the same Conscientiousness domain. The correlations were moderate enough to suggest that Grit’s facets are distinct from the Conscientiousness facets of the BFI-2, with one exception: Grit–Perseverance was essentially identical to BFI-2 Productiveness. Second, Grit’s facets show largely similar associations with external criteria as the three Conscientiousness facets. Ever so slightly, Grit’s facets had some differential and somewhat stronger associations especially with income and educational attainment. Third, Grit–Consistency, not Grit–Perseverance, emerged as the facet of Grit that appeared more distinct from other Conscientiousness facets and had mostly equally strong associations with the external criteria as Grit–Perseverance did. We elaborate on these findings below.

### Locating Grit’s facets in the facet structure of BFI-2 Conscientiousness

How are the two facets of Grit related to other facets of Conscientiousness? Our results showed the expected strong relationships between the two facets of Grit and the three facets of BFI-2 Conscientiousness. In a head-to-head comparison, the expected strong relationships between the two first-order facet pairs Grit–Perseverance and Productiveness as well as Grit–Consistency and Responsibility emerged. When using the bifactor-(*S*–1) model that controls for the variance these four facets share with Organization (the reference facet), the residual facets again showed the expected, and even stronger, partial relationships to each other.

All in all, the relationships we found add to earlier findings that attest to the close relationship between Grit and Conscientiousness using a broader measure to assess Conscientiousness than Schmidt et al. [8] and investigating the relationship on the facet level other than Credé et al. [7]. Our findings reflect the theoretically derived differential associations between the two facet pairs. Especially the near-perfect relationship between Grit–Perseverance and Productiveness (with the amount of shared variance approaching 90% in both models) stand in accordance with the findings by Schmidt et al. [8] and call into question whether Grit–Perseverance is empirically distinguishable from Productiveness. They stress the possibility that Grit–Perseverance is just a redundant re-labeling of a well-established Conscientiousness facet, rather than an substantial contribution to the canon of constructs in psychology. Again as earlier findings suggested [8, 18], a substantial amount of unique variance still pertained to residual Grit–Consistency, even though it was highly correlated with the Responsibility facet of the BFI-2, to which it is conceptually most closely related (62% shared variance). This led us to conclude that, even though theoretically similar, Grit–Consistency did not show a complete overlap with Responsibility and does capture something unique that the three Conscientiousness facets do not capture. As a glance at the items in S1 Table suggest, Grit–Consistency captures the ability to adhere to a given goal over longer periods of time more explicitly than the other facets do. These results suggest that Grit–Consistency, but not Grit–Perseverance, may contain information that is not captured by the three established Conscientiousness domains of the BFI-2.

### Correlations with external criteria and incremental value

How are the facets of Grit and Conscientiousness related to external criteria? We investigated the relationships with relevant external criteria to probe the added value of Grit’s facets over those of Conscientiousness. Despite the close relationship between some of the facets, the correlational pattern was nuanced. In the head-to-head comparison of the relationships that the facets of Grit and Conscientiousness had to external criteria, similar relationships emerged, except for educational attainment and the intelligence measures (Gf, Gc). Although the first-order Grit facets are related closely to the first-order Conscientiousness facets, only the first-order Grit facets showed statistically significant correlations with educational attainment, thereby corroborating to the assumption that Grit captures aspects of Conscientiousness that are related to success in academic settings. Across the full set of criteria we investigated, neither of the five facets emerged as clearly superior over the others in terms of their associations with these criteria.

The correlations with fluid intelligence in Model A corroborated some of the earlier findings on the non-significant relationship between fluid intelligence and Grit [1, 28, 32]. The first-order Grit facets did not show a statistically significant relationship, whereas the first-order facets Organization and Productiveness did. In addition, the relationship between crystallized intelligence and Grit was investigated. The positive relationship that we found seems plausible [57]: other than fluid intelligence that can be understood as innate cognitive ability, crystallized intelligence is a result of long term investment of effort, an aspect that is at the core of the Grit construct. However, further investigations are needed to investigate the relationship in depth.

Previous research suggested that Grit–Perseverance outdoes Grit–Consistency (e.g., [7]) in terms of criterion validity. The present research does not. How can this difference be explained? The present research analyzed Conscientiousness on a facet level and revealed that that Grit–Perseverance is highly similar to the BFI-2 Conscientiousness facet Productiveness (90% shared variance). Previous research did not explicitly measure this specific Conscientiousness facet and thus may have overestimated the effect of Grit–Perseverance compared to Grit–Consistency. All in all, our findings corroborate the view that the two facets of Grit function similarly, albeit in a more complex manner than previously suggested.

Now we turn to the question of whether the facets of Grit show *incremental* relationships over BFI-2 Conscientiousness. The associations with external criteria of the two Grit facets were largely similar in direction and size to those of the three Conscientiousness facets with these criteria. However, there was a tendency in both Models A and B for Grit–Perseverance to show slightly stronger associations with the indicators of life success and performance—education, income, life satisfaction, and health—than all other facets. Moreover, the two facets of Grit showed slightly smaller associations with fluid intelligence than those of Conscientiousness did.

Even though both facets of Grit hold value over Conscientiousness referenced by the Organization facet of the BFI-2, the findings of the present investigation show important differences in the relationships with the Conscientiousness facets and the external criteria. Grit–Perseverance on the one hand showed a near complete overlap with the Productiveness facet of Conscientiousness and showed to be of incremental value over the referenced facet in Model B. These findings lead us to suggest, that Grit–Perseverance rather is a pure measure of the Productiveness or Industriousness facet of Conscientiousness, similar to the findings by Schmidt et al. [8]. We therefore do not contest the notion that Grit–Perseverance falls victim of a jangle fallacy.

Grit–Consistency, on the other hand, did share less variance with the Responsibility facet of Conscientiousness and showed to stand in relationship with all of the external criteria we used in this study over the referenced facet in Model B. These findings indicate, that Grit–Consistency reflects an aspect of Conscientiousness that is not captured fully by the BFI-2 (or the NEO-PI-R; [8]) and at the same time holds value over BFI-2 Conscientiousness. Up until now, it is still unclear if Grit–Consistency (1) reflects an aspect of Conscientiousness that is simply not reflected by the measures used to assess Conscientiousness in previous research, (2) it reflects a novel aspect of Conscientiousness that contributes to the personality domain, potentially due to the long-term aspect of the facet, and thus should be added to the conceptions of Conscientiousness such as the hierarchical structure of Conscientiousness by Roberts and colleagues [20], or (3) Grit–Consistency lends aspects from other domains of personality research or even constructs apart from personality research such as goal theory [72]. If so, Grit–Perseverance and Grit–Consistency would indeed be sufficiently distinct that an aggregation as done in the Grit conception would be questionable as argued recently by Credé [6].

In conclusion, our findings suggest that Grit–Perseverance adds little to the canon of constructs in the Conscientiousness domain but appears to be a scale with great utility to assess the proactive aspect of Conscientiousness, a domain of great relevance in educational psychology. Grit–Consistency, on the other hand, reflects aspects not fully captured by BFI-2 Conscientiousness, which proved to be of added value. Future research will show how to best categorize Grit–Consistency in the vast array of constructs and theories in psychological research.

Even though Grit and Conscientiousness are closely related, researchers argue that Conscientiousness is a distinct personality domain while Grit is a malleable personality trait. Up until now, some studies showed promising results regarding the malleability of Grit. Interventions to foster Grit through deliberate practice [73] or a growth mindset [74] showed at least some short-term effects. A recent study showed that a Grit intervention in elementary school led to long-term effects on goal-directed behavior and higher achievement in a math test follow-up after 2.5 years [75]. Whereas effects on achievement could be shown, the actual development of Grit over time was not tested in either study. Nonetheless, Grit might be malleable even if it is a personality trait in the Conscientiousness domain [1]. The neosocioanalytic model of personality trait development [76] may explain the development of Grit throughout life and through interventions. The model offers a theoretical explanation of the change of personality traits by identifying several mechanisms [77]. The theoretical approach that aims to explain how transitional experiences and situational demands may shape personality, commonly assumes that the changes in personality are preceded by behavioral changes. Following, environmental demands can create a reward structure that promotes self-regulated and consistent changes in behavior that in turn may cause changes in traits [78]. These considerations are in accordance with the approaches used in the studies described above and are of high relevance in regard to the presumed malleability of Grit. To test whether Grit and its facets might be more (or less) malleable than Conscientiousness was beyond the scope of the present investigation. We would like to encourage further research in that domain.

### Limitations and directions for future research

Our study has some limitations that future research should address. On a theoretical level, the key distinction between Grit and Conscientiousness is that Grit emphasizes the *long-term* perseverance of effort and consistency of interest more than most measures of Conscientiousness do. Although our results suggest that the items from Duckworth’s [1] original Grit scale–especially those intended to measure Grit–Perseverance–are not sufficiently distinct from Conscientiousness facets, this does not mean that constructing more distinct measurement instruments is impossible in principle. If the *long-term* aspect of Grit is what distinguishes it from Conscientiousness, it might be feasible to construct items that more explicitly reflect long-term goal Grit–Perseverance and Grit–Consistency. To investigate the long-term aspects of Grit and Conscientiousness, longitudinal study designs—preferably integrating the achievement of long-term goals—that surpass the currently available research using mostly retrospective assessments of past achievements are needed.

In addition, the cross-sectional design of our study prevents us from to drawing causal inferences regarding the relationships between Grit, Conscientiousness, and the external criteria. As we noted, the direction of causal influence was of minor import for our study. However, especially if Grit is to be used for selection and placement decisions, it will be important for future research to gauge the causal effects of Grit especially on success measures (e.g., job performance). Furthermore, research shows that Grit is malleable to a degree, a quality that is not necessarily part of the Conscientiousness conception, and, may be the central differentiating factor between the two. With our cross-sectional study design, we were not able to investigate the relative temporal stability and malleability of Grit in comparison to Conscientiousness. We would like to encourage future research to investigate this unresolved research question.

Moreover, we only used self-report measures to assess Grit, Conscientiousness, and external criteria, the limits of which are well documented [79]. To use other-ratings or more objective measures such as observer rating forms as proposed by Credé [6] in addition to self-report data could lower the possible common method bias, which cannot be ruled out in the present study.

Another limitation is that some of the external criteria in our study were only measured with single-item scales. Single-item measures may be less reliable (compared to actual or hypothetical multi-item scales), which may in turn attenuate criterion correlations. It is important to note that the methodologies to assess the reliability of single-item measures require to make assumptions beforehand and therefore statements on *the* reliability are typically challenging [80]. With these restrictions in mind, research shows high reliability (>.90) for single-item questions regarding age, education and income, attenuation bias thus will be small [81, 82]. Numerous studies found at least satisfactory reliability coefficients of the single-item measures used to assess life-satisfaction [83–86] and indicators for criterion validity similar to multi-item measures [84, 86, 87].

Research on the reliability of single-item measures of subjective well-being are scarcer. However, Stewart, Hays, and Ware [88]argue that short to single measures of subjective physical and mental health are useful in larger samples. One previous study showed that the reliability and criterion validity of single-item measures to assess subjective health are satisfactory [84], whereby another study showed satisfactory results for a single-item measure but to a lesser degree in comparison to multi-item measures [89]. All in all, previous research shows satisfactory results for the reliability and criterion validity of single-item measures to assess life satisfaction. The results with regard to subjective health must be treated with more caution due to the fewer and more heterogeneous findings. However, it is crucial to note that this attenuation bias would be exactly the same for the Grit and Conscientiousness facets. Our findings regarding the differential associations of the facets with external correlates would thus be unaffected by attenuation bias. However, future research is needed to confirm these assumptions.

Earlier studies showed that domain-specific measures to assess Grit such as academic Grit [28, 90] can give a more in depth understanding of the construct. We would like to encourage future research to expand our findings on the relationships between Grit and indicators of life success with a domain-specific approach to Grit.

## Conclusion

Is Grit just “old wine in new bottles”, a case of the jangle fallacy, as some critics have claimed [7, 91]? Our study supports a more nuanced view, suggesting that Grit might best be considered as “some new wine in an old bottle”: Grit’s two facets—perseverance of effort and consistency of interest—can be conveniently subsumed under the broader Conscientiousness domain. Whereas Grit–Perseverance proved to be essentially interchangeable with Productiveness (but not with Organization or Responsibility), Grit–Consistency was empirically distinct from Grit–Perseverance and the three facets of Conscientiousness. That is, especially Grit–Consistency captures something more than Grit–Perseverance and other facets of Conscientiousness do.

In terms of their relationships with the external criteria, the two facets of Grit showed similar associations as the three facets of Conscientiousness did, with neither facet emerging as universally superior over the others. However, the pattern of correlations suggested that Grit’s facets tend to be more strongly related with criteria that are likely to reflect success after long-term investments of effort (i.e., income, crystallized intelligence, and–although here reciprocal effect are likely–educational attainment) than the facets of Conscientiousness. Together with the presence of some differential relationships with some of the external criteria, this suggests that differentiating Grit’s facets from each other, as well as from other facets of Conscientiousness, may be worthwhile when investigating the relationships with success measures. Moreover, as an anonymous reviewer pointed out, even if Grit offers little added value over existing measures of Conscientiousness, the label “Grit” might be a catchier term that is more readily understood by practitioners and policymakers than “Conscientiousness” or “Perseverance”. From this perspective, the fact that the Grit label is partly a jangle fallacy could be balanced against the benefits this label confers for the effective communication of research findings.

All in all, our findings give further purchase to the emerging consensus that Grit and its facets are best viewed as facets of the broader Conscientiousness domain. They show that Grit–Consistency, but not Grit–Perseverance, contains trait information that is not fully captured by the three Conscientiousness facets of the BFI-2. At the same time, and perhaps surprisingly, it is Grit–Consistency that stands out in terms of its relationships with criteria that index success after long-term investment of effort. More generally, our findings attest to the insights that can be gained from a facet-level perspective, as opposed to investigating compound measures at the domain level.

## Supporting information

S1 Table English version of items used in the study. BFI-2 items copyright 2016 by Oliver P. John and Christopher J. Soto.
(DOCX)

S2 Table German version of items used in the study. BFI-2 items copyright 2016 by Oliver P. John and Christopher J. Soto.
(DOCX)

S3 Table Descriptive statistics and correlations between the external criteria used in this study. *p < .05, **p < .001. Gf = Fluid intelligence. Gc = Crystallized intelligence. LS = Life satisfaction.
(DOCX)
