# Supplementary material for: New wine in an old bottle? A facet-level perspective on the added value of Grit over BFI–2 Conscientiousness
Source: PLoS One. 2020 Feb 13;15(2):e0228969. doi: 10.1371/journal.pone.0228969 (PMC7018017; doi:10.1371/journal.pone.0228969)
Supplement: S1 Table — BFI-2 items copyright 2016 by Oliver P. John and Christopher J. Soto. (DOCX) [file pone.0228969.s001.docx]

# Supplemental online material

Table S1

*English Version of Items Used in the Study*

| Facet | No. | English Version |
| --- | --- | --- |
| Conscientiousness |  | I am someone who... |
| Organization | 1 | tends to be disorganized. |
|  | 2 | is systematic, likes to keep things in order. |
|  | 3 | keeps things neat and tidy. |
|  | 4 | leaves a mess, doesn’t clean up. |
| Productiveness | 5 | is efficient, gets things done. |
|  | 6 | is persistent, works until the task is finished. |
|  | 7 | tends to be lazy. |
|  | 8 | has difficulty getting started on tasks. |
| Responsibility | 9 | can be somewhat careless. |
|  | 10 | sometimes behaves irresponsibly. |
|  | 11 | is reliable, can always be counted on. |
|  | 12 | is dependable, steady. |
| Grit |  |  |
| Perseverance | 13 | I am a hard worker. |
|  | 14 | Setbacks don’t discourage me. |
|  | 15 | I finish whatever I begin. |
| Consistency | 16 | I often set a goal but later choose to pursue a different one. |
|  | 17 | New ideas and new projects sometimes distract me from previous ones. |
|  | 18 | I have difficulty maintaining my focus on projects that take more than a few month to complete. |

Note. BFI-2 items copyright 2016 by Oliver P. John and Christopher J. Soto.
