# Supplementary material for: New wine in an old bottle? A facet-level perspective on the added value of Grit over BFI–2 Conscientiousness
Source: PLoS One. 2020 Feb 13;15(2):e0228969. doi: 10.1371/journal.pone.0228969 (PMC7018017; doi:10.1371/journal.pone.0228969)
Supplement: S2 Table — BFI-2 items copyright 2016 by Oliver P. John and Christopher J. Soto. (DOCX) [file pone.0228969.s002.docx]

Table S2

*German Version of Items Used in the Study*

| Facet | No. | German Version |
| --- | --- | --- |
| Conscientiousness |  |  |
| Organization | 1 | Ich bin eher unordentlich. |
|  | 2 | Ich bin systematisch, halte meine Sachen in Ordnung. |
|  | 3 | Ich mag es sauber und aufgeräumt. |
|  | 4 | Ich bin eher der chaotische Typ, mache selten sauber. |
| Productiveness | 5 | Ich bin effizient, erledige Dinge schnell. |
|  | 6 | Ich bleibe an einer Aufgabe dran, bis sie erledigt ist. |
|  | 7 | Ich bin bequem, neige zu Faulheit. |
|  | 8 | Ich neige dazu, Aufgaben vor mir herzuschieben. |
| Responsibility | 9 | Ich bin manchmal ziemlich nachlässig. |
|  | 10 | Manchmal verhalte ich mich verantwortungslos, leichtsinnig. |
|  | 11 | Ich bin verlässlich, auf mich kann man zählen. |
|  | 12 | Ich bin stetig, beständig. |
| Grit |  |  |
| Perseverance | 13 | Ich bin jemand, der hart arbeitet. |
|  | 14 | Ich komme mit Rückschlägen gut zurecht. |
|  | 15 | Was ich begonnen habe, bringe ich auch zu Ende. |
| Consistency | 16 | Ich kann mich kurzfristig für Ideen begeistern, verliere aber dann das Interesse daran. |
|  | 17 | In der Verfolgung meiner Ziele lasse ich mich manchmal von anderen Ideen ablenken. |
|  | 18 | Ich habe Schwierigkeiten, meine Konzentration auf Vorhaben oder Aufgaben aufrecht zu erhalten, deren Durchführung mehr als ein paar Monate in Anspruch nimmt. |

Note. BFI-2 items copyright 2016 by Oliver P. John and Christopher J. Soto.
