# Supplementary material for: New wine in an old bottle? A facet-level perspective on the added value of Grit over BFI–2 Conscientiousness
Source: PLoS One. 2020 Feb 13;15(2):e0228969. doi: 10.1371/journal.pone.0228969 (PMC7018017; doi:10.1371/journal.pone.0228969)
Supplement: S3 Table — *p < .05, **p < .001. Gf = Fluid intelligence. Gc = Crystallized intelligence. LS = Life satisfaction. (DOCX) [file pone.0228969.s003.docx]

Table S3

*Descriptive Statistics and Correlations Between the External Criteria Used in This Study*

|  | Range | *M* | *SD* | 2. | 3. | 4. | 5. | 6. | 7. |
| --- | --- | --- | --- | --- | --- | --- | --- | --- | --- |
| 1. Age | 18-65 | 43.34 | 13.97 | –.09** | .17** | .07* | –.19** | –.31** | .27** |
| 2. Education | 1-6 | 4.35 | 1.45 |  | .25** | .17** | .19** | .36** | .37** |
| 3. Income | 1-17 | 7.05 | 3.56 |  |  | .21** | .19** | .00 | .17** |
| 4. LS | 1-11 | 7.17 | 2.47 |  |  |  | .48** | .02 | .10 |
| 5. Health | 1-5 | 3.23 | 1.10 |  |  |  |  | .08 | .07 |
| 6. Gf | 0-1 | .34 | .26 |  |  |  |  |  | .28** |
| 7. Gc | 0-1 | .66 | .21 |  |  |  |  |  |  |

*Note.* **p* < .05, ***p* < .001. Gf = Fluid intelligence. Gc = Crystallized intelligence. LS = Life satisfaction.
